# Relation of Posterior Cerebellar Volume to Cognition in MS

**DOI:** 10.15844/pedneurbriefs-29-7-4

**Published:** 2015-07

**Authors:** J. Gordon Millichap

**Affiliations:** 1 Division of Neurology, Ann & Robert H. Lurie Children's Hospital of Chicago, Chicago, IL; 2 Departments of Pediatrics and Neurology, Northwestern University Feinberg School of Medicine, Chicago, IL

**Keywords:** Cerebellum, Cognition, Multiple Sclerosis, Pediatric, Volumetric MRI

## Abstract

Investigators from the Montreal Neurological Institute, York University, Universities of Toronto and McGill, Canada, and University of Pennsylvania, studied the relationship between cerebellar pathology and cognitive function in adolescent and pediatric-onset multiple sclerosis (MS).

Investigators from the Montreal Neurological Institute, York University, Universities of Toronto and McGill, Canada, and University of Pennsylvania, studied the relationship between cerebellar pathology and cognitive function in adolescent and pediatric-onset multiple sclerosis (MS). Twenty-eight pediatric-onset relapsing remitting MS patients (21 girls, mean age 16.2 years; mean disease duration 4.3 years) were compared to 33 age- and sex-matched healthy controls. Participants underwent structural MRI and neuropsychological evaluation to assess intelligence, attention, processing speed, language visuo-motor integration, and fine motor dexterity. Associations between cognitive outcomes and cerebellar volume, independent of cerebral volume were examined.

Cognitive and motor performance of the MS group was reduced relative to controls (p<0.003). Cerebellar volumes did not differ between groups, but cerebellar posterior lobe volume and infratentorial lesion volume were correlated with extra variance on measures of information processing (p=0.02) and vocabulary (p=0.04) in patients, but not in controls. The investigators conclude that smaller cerebellar posterior lobe volume, a known region for cognitive processing and increased lesion burden in the posterior fossa, adversely impact cognitive function, an important functional consequence of MS onset during childhood. [1]

COMMENTARY. The cerebellum is involved in cognitive and affective processes in addition to motor function. Cerebellar motor and cognitive dysfunction occur in parallel early in the onset of MS. This association is well documented in adult MS but is less recognized in children with MS, highlighting differences between pediatric and adult cases. Language is particularly vulnerable in pediatric MS, unlike in adults in whom it is usually preserved. Deficits in executive functions considered MS-specific in adults, have been inconsistently reported in children. Data on the correlations of cognitive impairments with clinical and neuroimaging are scarce in children, and the results are often incongruent. Involvement of the corpus callosum and reduced thalamic volume differentiate patients with cognitive impairment from those without. [2]. Cerebellar posterior lobe volume and infratentorial lesion volume account for extra variance on measures of information processing and vocabulary in patients but not in controls. In a further study of the relation between cerebellar MS atrophy and cognitive dysfunction, patients with cerebellar dysfunction performed worse in cognitive tests than controls [3].
